# Case Report: Optimizing ICU management of sickle cell crisis: the impact of bedside ultrasound on clinical decision-making

**DOI:** 10.3389/fmed.2026.1804466

**Published:** 2026-04-15

**Authors:** Devanshi Mehta

**Affiliations:** Section of Pulmonary and Critical Care Medicine, Department of Medicine, The University of Texas at Tyler, Tyler, TX, United States

**Keywords:** acute chest syndrome, critical care echocardiography, Point-of-Care Ultrasound (POCUS), pulmonary hypertension, sickle cell crises

## Abstract

**Introduction:**

Acute chest syndrome (ACS) is a leading cause of mortality in Sickle Cell Disease (SCD), often characterized by rapid respiratory decline and acute pulmonary hypertension (PH). While exchange transfusion is the standard of care for severe cases, delayed access to hemoglobin (Hb) electrophoresis often hinders real-time monitoring of therapeutic efficacy. We propose that POCUS guided assessments of pulmonary arterial pressures via tricuspid regurgitation jet velocities can serve as a real-time hemodynamic tool to direct serial exchange transfusions thereby preventing right heart failure and mortality in severe acute chest syndrome.

**Case presentation:**

A 20-year-old male patient with HbSS (baseline HbS 28.7%, on hydroxyurea) presented with shortness of breath, severe hip/back pain and acute hemolysis (Hb 8.3 g/dL, bilirubin 7.4 mg/dL, LDH 484 U/L). Despite treatment for ACS and an initial exchange transfusion, his oxygen requirements escalated from simple nasal cannula to high-flow nasal cannula. Repeated imaging showed worsening infiltrates, and Point-of-Care Ultrasound (POCUS) revealed acute PH (TR jet velocity > 4 m/s).

**Management and outcome:**

Following the second exchange transfusion, the patient’s tachycardia, dyspnea, and oxygen requirements rapidly improved. Follow-up Point-of-Care Ultrasound (POCUS) demonstrated an improved and now trace tricuspid regurgitation. Subsequent electrophoresis confirmed the first exchange only reduced HbS to 49%, while the second achieved a therapeutic level of 20.9% (recommended target HbS of <30% by American Society of Hematology).

**Discussion/conclusion:**

This case demonstrates that acute elevations in pulmonary artery pressure can serve as a critical surrogate marker for ongoing sickling when electrophoresis results are delayed. The patient’s TRV of 3.74 m/s placed him in a high-mortality cohort (*P* < 0.001). Given that POCUS provides high diagnostic accuracy (AUC 0.87), it may be utilized as a real-time hemodynamic monitor to guide the necessity of serial exchange transfusions in the absence of immediate HbS% quantification. In severe ACS, achieving a target HbS ≤ 30% is vital, and bedside echocardiography may identify patients requiring immediate repeat exchange transfusion to prevent right heart failure and death.

## Introduction

Acute chest syndrome constitutes a form of lung injury in sickle cell disease. The underlying mechanisms include pulmonary infection, and pulmonary fat embolism, leading to lung injury, such as pulmonary infarction (1). This can lead to pulmonary hypertension through vaso-occlusion, hypoxic vasoconstriction, and endothelial dysfunction (1, 2), collectively increasing pulmonary vascular resistance and right ventricular afterload. These changes elevate the pressure gradient across the tricuspid valve, driving higher tricuspid regurgitant (TR) jet velocities that provides a non-invasive bedside estimate of pulmonary artery systolic pressure (3). Bedside POCUS can detect tricuspid regurgitation and measure TR jet velocities, with a resting value of 2.9 m/s or greater indicating elevated pulmonary pressures and right heart strain. Ultrasound also facilitates rapid bedside diagnosis of pneumonia, as indicated by dynamic air bronchograms and consolidations (4), and pulmonary infarction, as evidenced by the survived lung sign and absence of blood flow on color Doppler (5).

The American Society of Hematology recommends exchange transfusion for acute chest syndrome in severe or rapidly progressive cases, with a target HbS percentage of less than or equal to 30%. Hemoglobin electrophoresis results may not be immediately available for decision-making in critically ill patients with acute chest syndrome. Acute elevation in TR jet velocities may serve as a clinically actionable surrogate marker of refractory sickling when electrophoresis is delayed, highlighting the utility of POCUS in guiding timely, life-saving decisions regarding serial exchange transfusions in severe acute chest syndrome.

## Case presentation

A 20-year-old man with HbSS and a history of recurrent vaso-occlusive crises and acute chest syndrome was admitted for severe hip and lower back pain. He had been maintained on hydroxyurea since adolescence, with a baseline dose of 2,000 mg daily.

On admission, he was normotensive but markedly tachycardic (heart rate 120–140 beats/min, sinus rhythm). Laboratory studies revealed hemoglobin 8.3 g/dL (normal 14–18 g/dL), total bilirubin 7.4 mg/dL (normal 0.3- 1mg/dL) and indirect bilirubin 6.2 mg/dL, LDH 484 U/L (normal 107- 249 U/L), and reticulocyte count 13.8% (normal 0.5- 2.5%), consistent with active hemolysis. Chest X ray on initial presentation demonstrates multifocal infiltrates, a retrocardiac air bronchogram as silhouette sign with obscuration of the right heart border consistent with early ACS. Oxygen saturation was >90% on room air at rest. He was treated with aggressive hydration, analgesia, empiric broad-spectrum antibiotics, and continuation of home hydroxyurea. A temporary dialysis catheter was placed in anticipation of exchange transfusion.

Over the subsequent 24 h, his oxygen requirement escalated from room air to 6 L/min via nasal cannula. He underwent exchange transfusion on hospital day 2, with post-exchange hemoglobin electrophoresis ordered. Despite aggressive care—including diuresis and high-flow oxygen—he developed progressive hypoxemia and persistent sinus tachycardia. Repeat chest radiography demonstrated worsening bilateral infiltrates.

**FIGURE 1:**
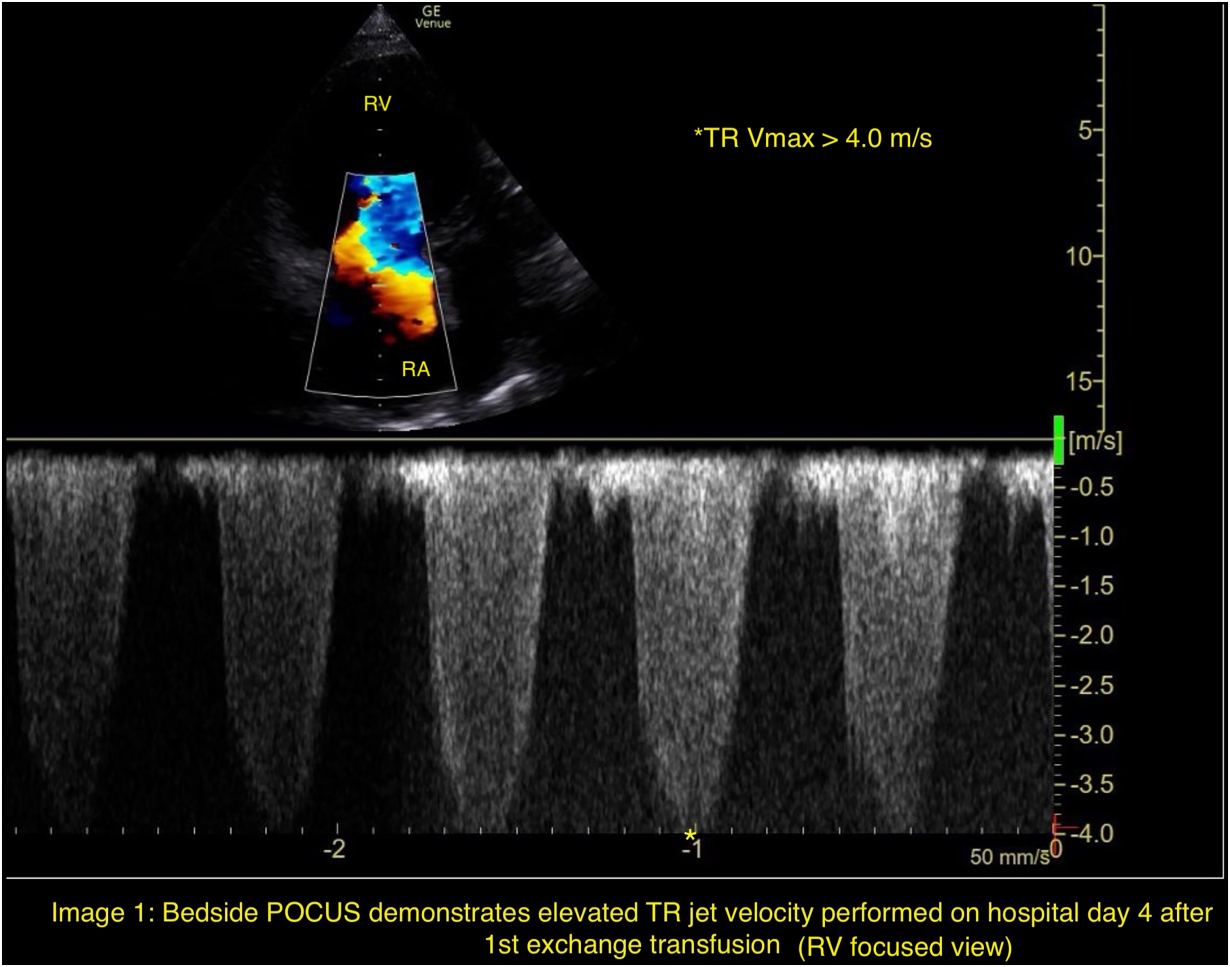
Bedside POCUS demonstrates elevated TR jet velocity performed on hospital day 4 after 1st exchange transfusion (RV focused view).

**FIGURE 2:**
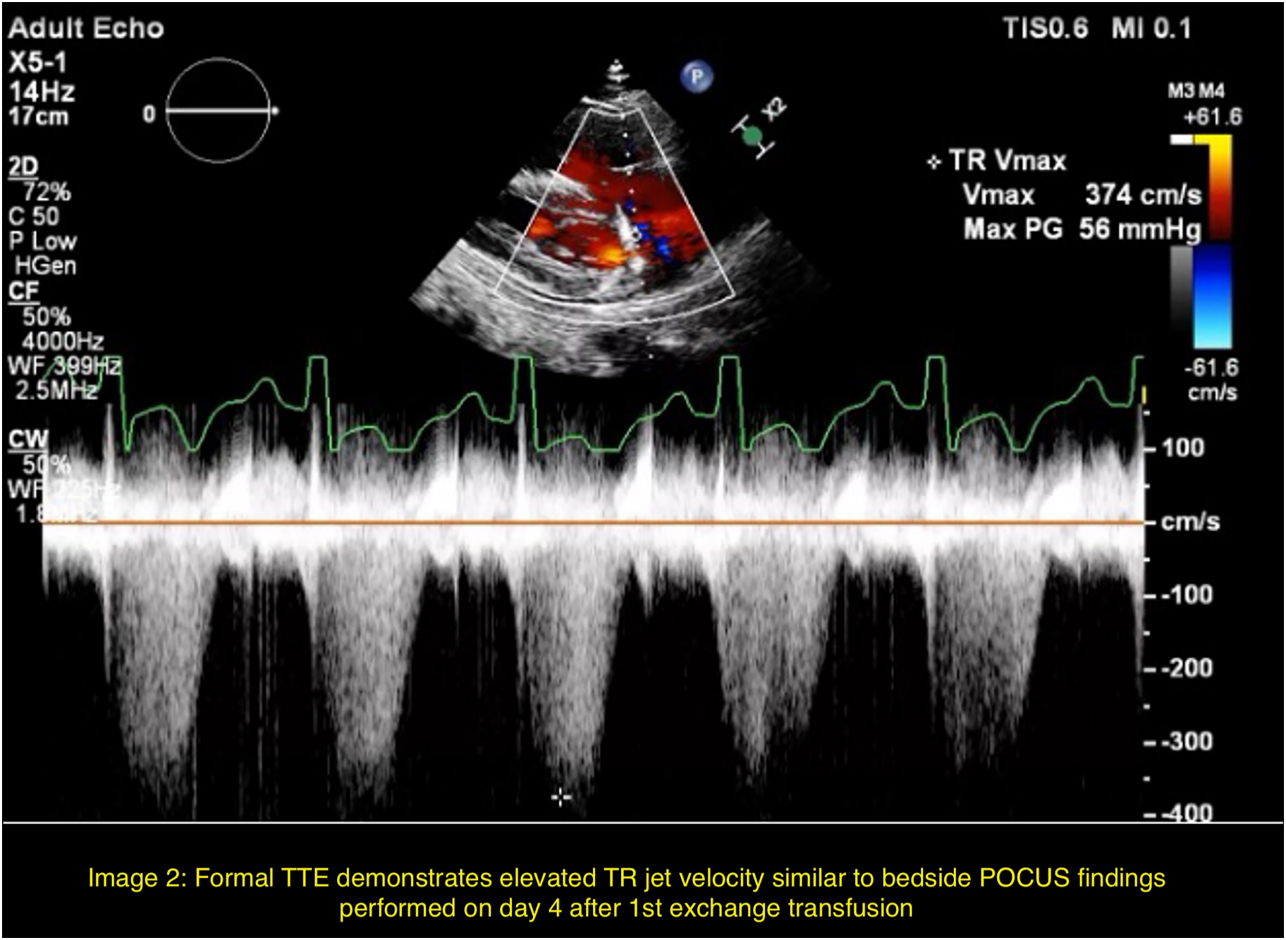
Formal TTE demonstrates elevated TR jet velocity similar to bedside POCUS findings performed on day 4 after 1st exchange transfusion.

Bedside point- of- care ultrasound (POCUS) on hospital day 4 revealed elevated right-sided pressures, reflected by severe tricuspid regurgitation with increased tricuspid regurgitant jet velocity > 4.0 m/s and end-systolic flattening of the interventricular septum (Figure 1). This was confirmed by a formal transthoracic echocardiogram subsequently performed on hospital day 4 demonstrating acute pulmonary hypertension and a tricuspid regurgitant jet velocity of 3.73 m/s (Figure 2). Concurrent laboratory evaluation showed worsening hemolysis (hemoglobin 8.1 g/dL, total bilirubin 12.2 mg/dL, LDH 696 U/L); direct antiglobulin testing was negative. Hemoglobin electrophoresis results from admission and after the first exchange were still pending.

**FIGURE 3:**
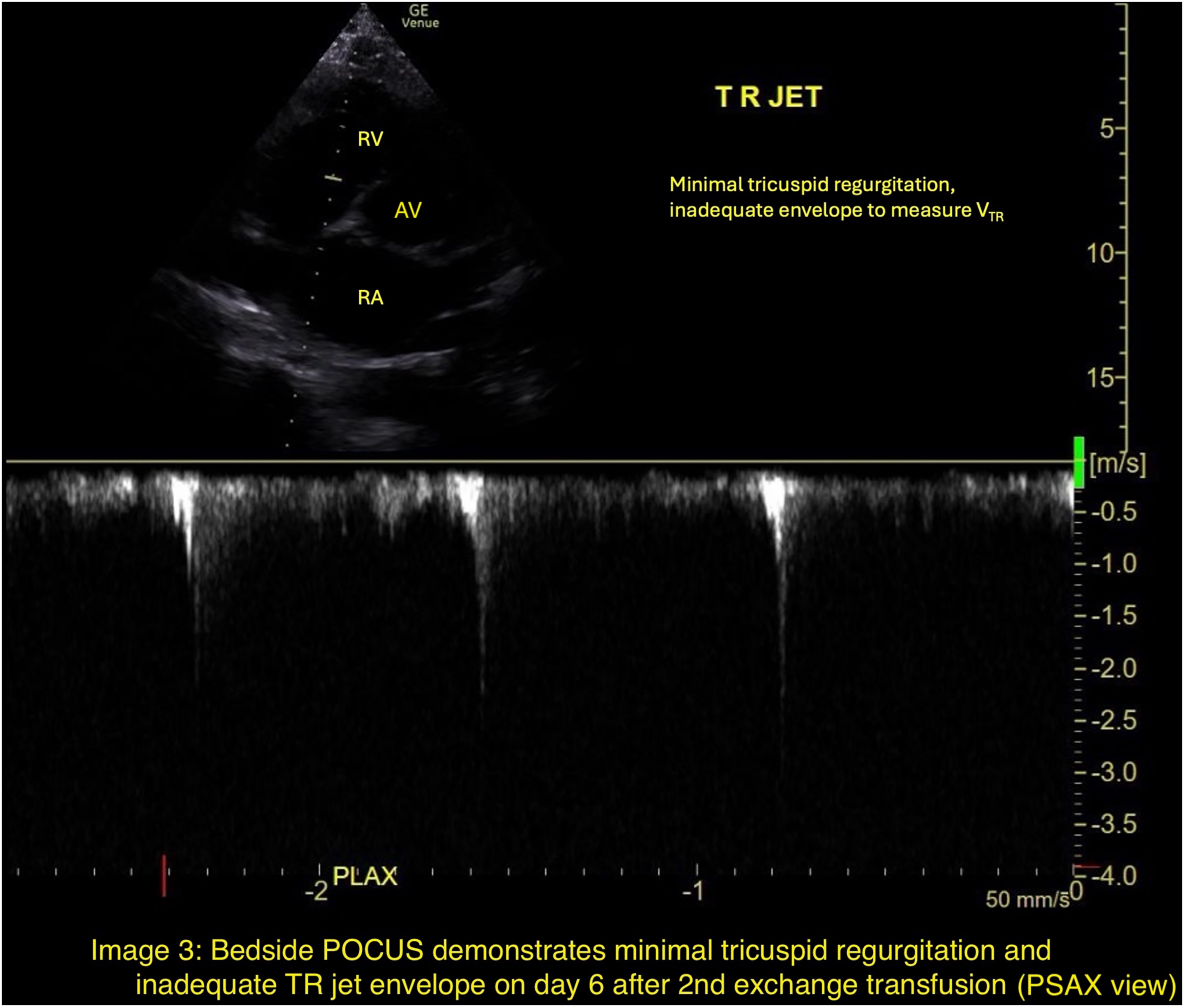
Bedside POCUS demonstrates minimal tricuspid regurgitation and inadequate TR jet envelope on day 6 after 2nd exchange transfusion (PSAX view).

**FIGURE 4:**
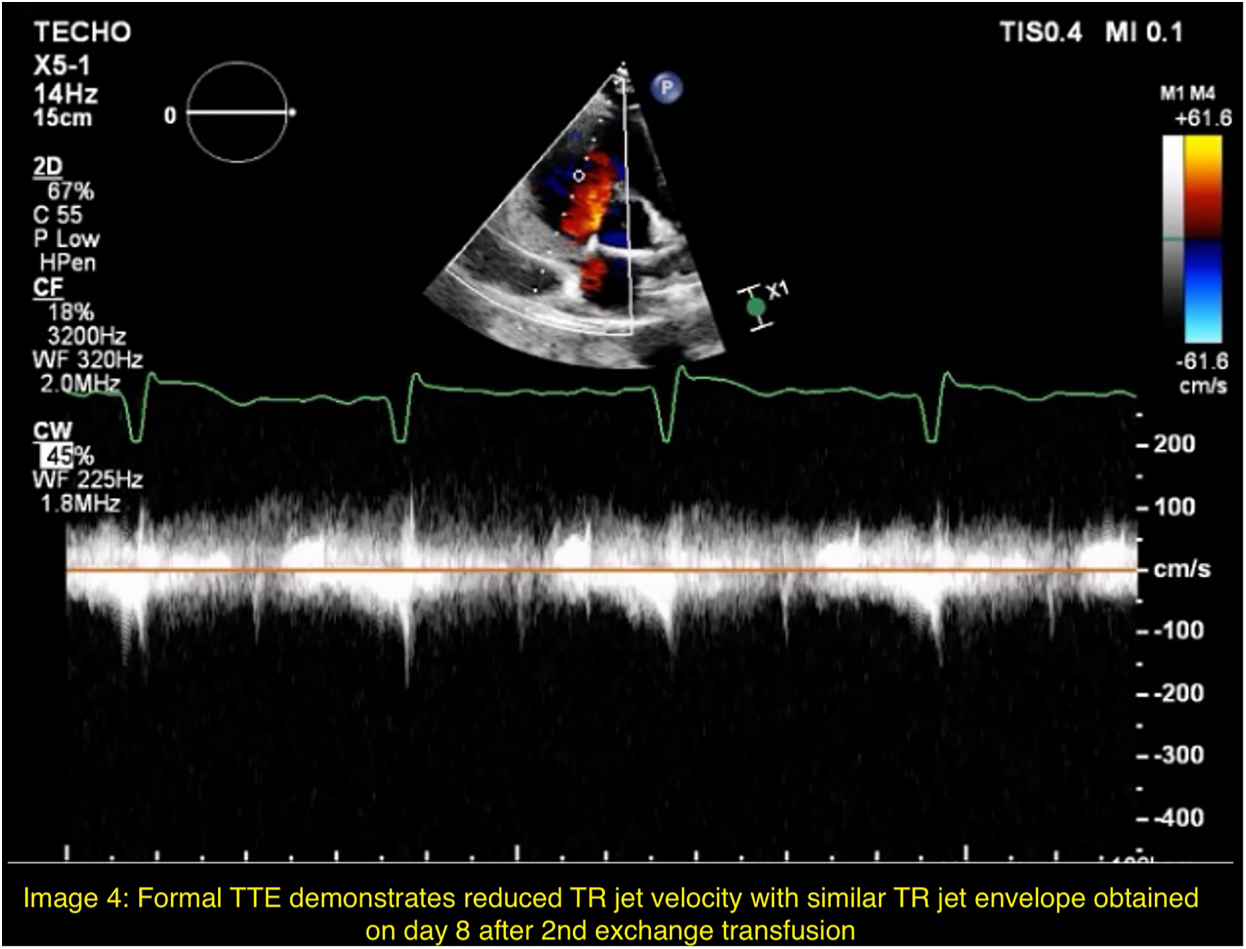
Formal TTE demonstrates reduced TR jet velocity with similar TR jet envelope obtained on day 8 after 2nd exchange transfusion.

Given persistent hypoxemia, worsening hemolysis, and evidence of acute pulmonary hypertension concerning for ongoing sickling, a second exchange transfusion was performed on hospital day 5. The patient demonstrated rapid improvement in oxygenation, heart rate, and dyspnea over the next 24 h. Follow-up chest radiography showed interval improvement. Trace tricuspid regurgitation with inadequate TR jet envelope was seen on POCUS on hospital day 6 and TR jet velocity could not be measured (Figure 3). A repeat formal transthoracic echocardiogram performed on hospital day 8 demonstrated normalization of pulmonary pressures, with tricuspid regurgitant jet velocity reduced to 2.08 m/s (Figure 4). He was gradually weaned from high-flow nasal cannula to standard nasal cannula by hospital day 10 and transferred out of the ICU. Hemoglobin electrophoresis results became available sequentially and confirmed an initial HbS fraction of 80.8%, an inadequate reduction following the first exchange (49%), and achievement of therapeutic targets after the second exchange (20.9%).

## Investigations

At baseline, patient’s Hb level varies between 9 and 10 g/dL, with a hematocrit of 27.3% and reticulocyte percentage of 3.47. Most recent Hb electrophoresis (prior to hospital admission obtained on outpatient basis) demonstrates HbS fraction of 28.7% and HbF of 6%. Hb electrophoresis at our institution is performed by capillary electrophoresis with high accuracy of 93- 100% (1, 6, 7). The turnaround time for this test is high (3–5 days) and HbS% is often not available for decision making. The patient had undergone an outpatient transthoracic echocardiogram about a year prior to this admission which demonstrated normal RV function, with trace tricuspid regurgitation and TR jet velocity could not be measured due to inadequate doppler signal strength. His chest x-ray on initial presentation demonstrates multifocal infiltrates, a retrocardiac air bronchogram as silhouette sign with obscuration of the right heart border. Subsequently his chest x ray worsened, and his supplemental oxygen requirements increased.

**Table:** 

**Pertinent laboratory and echocardiographic values**				
**Laboratory values** **(reference values) units**	**Baseline**	**Presentation/ Day 1**	**Post 1^st^ ET**	**Post 2^nd^ ET**
Hb (14–18) g/dL	10.22	8.1	8.1	7.8
Hct (38–54)%	27.3	22.8	22.7	22.3
Reticulocyte (0.5–2.5)%	3.47	13.8	9.20	3.87
Total bilirubin (0.3–1) mg/dL	3.9	5.8	12.2	6.9
Direct bilirubin (0.03–0.18) mg/dL	–	1.22	–	–
LDH (107–249) U/L	–	484	696	593
Haptoglobin (30–200) mg/dL	<20	–	52	–
Coombs Test	–	–	Negative	–
Hb electrophoresis				
HbA1 (95–98.5)%	62.7	0.0	37	72
HbA2(1.6–3.7)%	32.6	2.4	2.6	2.5
HbF (0–2)%	6.0	16.8	11.4	4.3
HbS (none)%	28.7	80.8	49.0	20.9
Procalcitonin ng/mL	–	2.16	–	–
**Echocardiographic Values**	**Baseline (outpatient)**		**Post 1^st^ ET**	**Post 2^nd^ ET**
RV function	Normal		Moderate to severely dilated	Normal
Tricuspid regurgitation (if present)	Trace		Moderate TR	Mild
TR jet velocity m/s	Could not be measured due to inadequate doppler signal		3.74	2.08

## Discussion

Acute chest syndrome is a leading cause of mortality in individuals with sickle cell anemia (6). The prevalence of pulmonary hypertension within this population was identified to be 32% (8). Patients with both sickle cell anemia and pulmonary hypertension experience significantly higher mortality rates than those without pulmonary hypertension (8).

The mechanisms underlying increased vascular resistance and elevated pulmonary pressures in acute chest syndrome include the following:

(1) Nitric oxide scavenging by plasma free hemoglobin released from lysed red blood cells, which promotes vasoconstriction and microvascular thrombosis (1).
(2) Erythrocyte arginase mediated lowering of L- arginine levels (1) and
(3) Hypercoagulable state resulting from combined nitric oxide and arginine depletion (1, 9)

No specific treatments are recommended for elevated pulmonary artery pressures during sickle cell crises. Current recommendations for management of acute chest syndrome include transfusion (simple and exchange), antibiotics, hydration, oxygen supplementation, incentive spirometry, and bronchodilators (10, 11). The American Society of Hematology recommends simple or exchange transfusion for acute chest syndrome, with exchange transfusion reserved for severe or rapidly progressive cases or those requiring invasive ventilatory support (10). The primary objective of exchange transfusion is to reduce the concentration of sickling cells, targeting an HbS percentage of 30% or less. Hemoglobin electrophoresis results may not be immediately available for clinical decision-making in patients with acute chest syndrome. Therefore, alternative markers of elevated HbS percentage and ongoing sickling should be investigated. Acute chest syndrome contributes to pulmonary hypertension by causing vaso-occlusion, hypoxic vasoconstriction, hemolysis-mediated nitric oxide depletion, and thrombosis, which together increase pulmonary vascular resistance and right ventricular afterload (1, 2). These effects raise the pressure gradient across the tricuspid valve, resulting in higher tricuspid regurgitant (TR) jet velocities that indicate the severity of acute pulmonary hypertension. The simplified Bernoulli equation (RVSP = 4 × TR velocity^2^ + RAP) allows TR velocity to serve as a non-invasive bedside estimate of pulmonary artery systolic pressure (3, 12). Echocardiographic evidence of acute elevations in tricuspid regurgitant (TR) jet velocities in acute chest syndrome may serve as a marker of ongoing sickling and indicate the need for further exchange transfusions (3). Dessap et al. examined acute elevations in pulmonary artery pressure in patients with acute chest syndrome and identified positive correlations between high TRV and elevated BNP, serum AST, total bilirubin, and serum creatinine (2). Additionally, 60% of patients with acute chest syndrome had TR jet velocities greater than 2.5 m/s, and TRV greater than 3.0 m/s was significantly associated with mortality (2). Patients with baseline pulmonary hypertension may develop acute elevations in pulmonary artery pressure, both of which may contribute to the high mortality observed in acute chest syndrome (13).

Formal transthoracic echocardiogram results interpreted by cardiologists may not be immediately available at centers managing patients with acute chest syndrome. Bedside echocardiography is increasingly integrated into critical care medicine training programs (14). Intensivists and pulmonary/critical care fellows have demonstrated accurate assessments of right ventricular function, with AUC values of 0.87 and 0.83, respectively, compared to formal transthoracic echocardiograms reviewed by cardiologists (15). In this case, bedside echocardiography yielded TR jet velocity values comparable to those obtained from formal transthoracic echocardiograms. Bedside assessments of right heart function are rapidly available for immediate clinical decision-making, unlike parameters such as hemoglobin electrophoresis. In the present patient, HbS percentage, which became available 3 days after the second exchange transfusion, remained elevated at 49%, exceeding the recommended target of less than 30% (10). Further research is required to clarify the role of acute elevations in TR jet velocity in the management of sickle cell crises.

## Data Availability

The original contributions presented in the study are included in this article/supplementary material, further inquiries can be directed to the corresponding author.
